# Analysis of Human Protein Replacement Stable Cell Lines Established using snoMEN-PR Vector

**DOI:** 10.1371/journal.pone.0062305

**Published:** 2013-04-25

**Authors:** Motoharu Ono, Kayo Yamada, Akinori Endo, Fabio Avolio, Angus I. Lamond

**Affiliations:** Centre for Gene Regulation and Expression, College of Life Sciences, University of Dundee, Dundee, United Kingdom; St. Georges University of London, United Kingdom

## Abstract

The study of the function of many human proteins is often hampered by technical limitations, such as cytotoxicity and phenotypes that result from overexpression of the protein of interest together with the endogenous version. Here we present the snoMEN (snoRNA Modulator of gene ExpressioN) vector technology for generating stable cell lines where expression of the endogenous protein can be reduced and replaced by an exogenous protein, such as a fluorescent protein (FP)-tagged version. SnoMEN are snoRNAs engineered to contain complementary sequences that can promote knock-down of targeted RNAs. We have established and characterised two such partial protein replacement human cell lines (snoMEN-PR). Quantitative mass spectrometry was used to analyse the specificity of knock-down and replacement at the protein level and also showed an increased pull-down efficiency of protein complexes containing exogenous, tagged proteins in the protein replacement cell lines, as compared with conventional co-expression strategies. The snoMEN approach facilitates the study of mammalian proteins, particularly those that have so far been difficult to investigate by exogenous expression and has wide applications in basic and applied gene-expression research.

## Introduction

**Figure 1:**
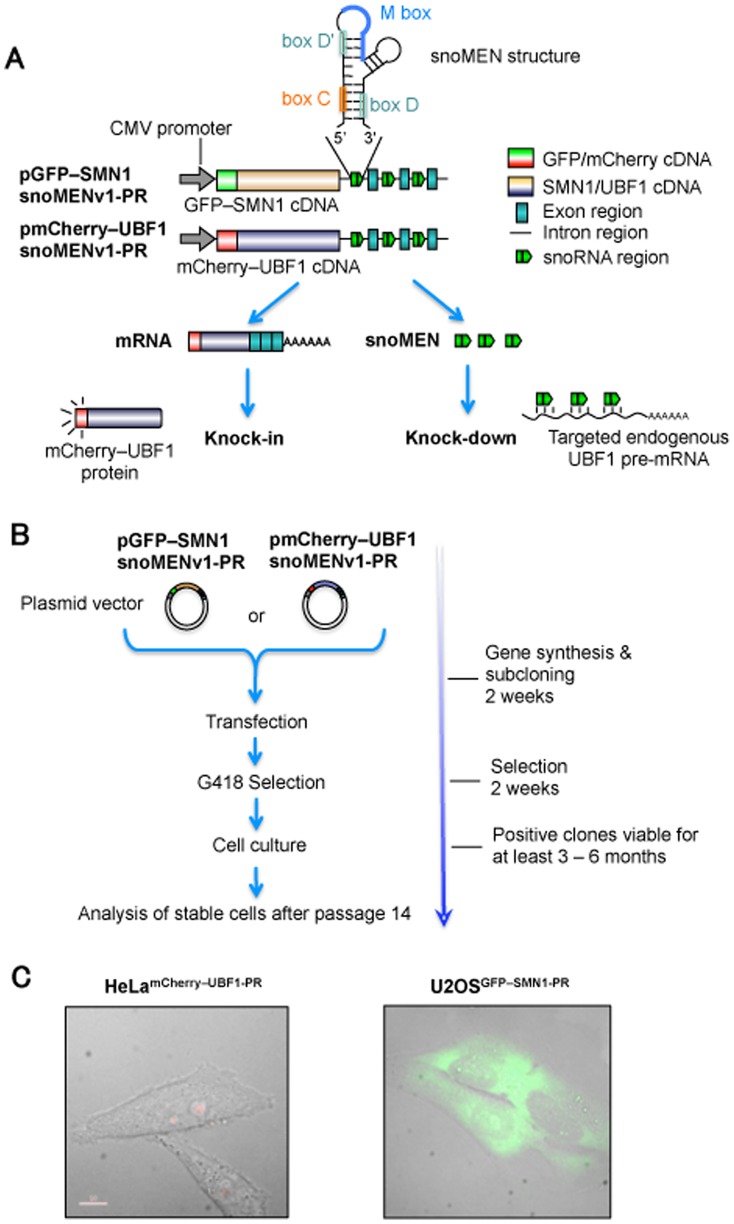
Procedure for establishment of human protein replacement stable cell line using snoMEN. (A) Structures for targeted endogenous SMN1/UBF1 protein replacement plasmids (pGFP–SMN1snoMENv1-PR/pmCherry–UBF1snoMENv1-PR). These constructs have three snoMEN sequences as previously described [1], except that the M box sequences are complementary to endogenous SMN1/UBF1 pre-mRNA sequences (See Materials and Methods). (B) Procedure of stable cell line establishment. Transfected cells were selected under G418 treatment as previously described (http://www.lamondlab.com/f7protocols.htm). Cells were cultured for at least 14 passages before analysis to confirm stable FP–protein and snoMEN expression. (C) Images of protein replacement stable cell lines. Expression of FP proteins was confirmed by fluorescence imaging. Bar length is 10 µm.

Methods for studying protein function often make use of either the transient or stable expression of an exogenous gene. In human cells this usually involves co-expression of the transgene together with the endogenous version. This often leads to overexpression, which can be toxic, and the presence of the endogenous protein can reduce the ability of the introduced protein to form complexes and interactions with cellular partners. To avoid these technical limitations we have created a vector system for the simultaneous targeted knock-down and replacement of endogenous proteins with exogenous tagged versions in mammalian cells. The snoMEN vector technology is based on the human box C/D small nucleolar RNA (snoRNA) HBII-180C, which contains an internal sequence (M box) that can be manipulated to make it complementary to RNA targets [1] (see Figure 1A).

SnoRNAs are an ancient class of conserved, nuclear non-coding RNAs (ncRNA) identified as guides for site-specific, post-transcriptional modifications in ribosomal RNA (rRNA) [2], [3], [4], [5]. Box C/D snoRNAs form functional complexes *in vivo* with small nucleolar ribonucleoproteins (snoRNPs), such as NOP56, NOP58, 15.5 K and the highly conserved protein fibrillarin, which is responsible for rRNA 2′-O-ribose methylation. The snoMEN methodology for the targeted modulation of gene expression is an antisense gene-suppression method with applications similar to siRNA and shRNA.

Major features of snoMEN technology that may differ from other knock-down systems are that a) snoMEN target nuclear RNAs, e.g. pre-mRNAs and non-coding RNAs, b) snoMEN RNAs are transcribed from RNA polymerase II promoters instead of the RNA polymerase III promoter required for shRNA plasmids, c) multiple snoMEN RNAs can be expressed within a single transcript under the regulation of a single promoter [1]. This allows the use of snoMEN technology for a wide variety of gene-regulation studies, including knock-down and/or knock-in analysis. The RNAi technologies based on siRNA, short hairpin RNA (shRNA), long double-stranded RNA (dsRNA), and microRNA (miRNA) function through assembly with cellular proteins to form an RNA-induced silencing complex (RISC) [6], [7], [8] and take advantage of the endogenous gene silencing machinery. Although the detailed snoMEN knock-down mechanism is still unknown, the snoMEN vector technology can also trigger targeted RNA degradation by a sequence-specific RNA:RNA base pairing event. SnoMEN can function in the nucleus and target pre-mRNA sequences, including intron sequences [1].

**Figure 2:**
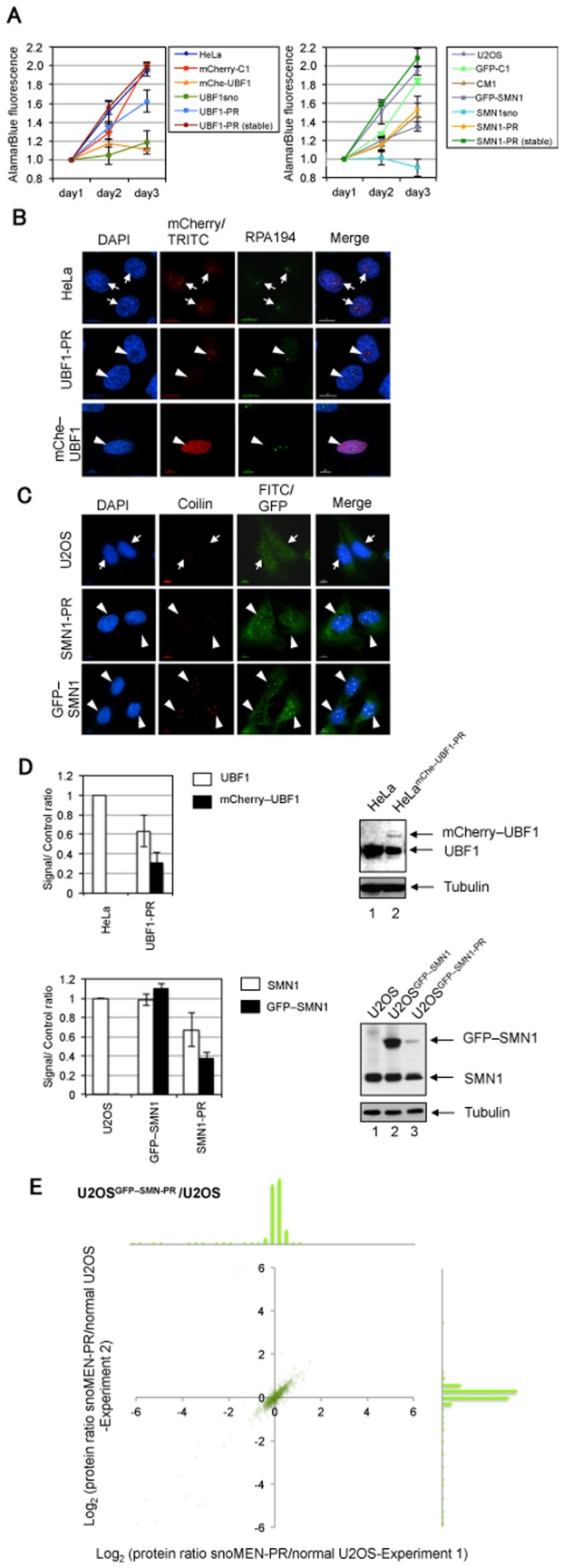
Characterisation of snoMEN protein replacement stable cell lines. (A) Results of proliferation/cytotoxicity assay for UBF1 (left panel) and SMN1 (right panel) protein replacement stable cell lines. Proliferation of the HeLa^mCherry–UBF1-PR^ [UBF1-PR (stable)] and U2OS^GFP–SMN1-PR^ [SMN-PR (stable)] cell lines were compared with their host HeLa and U2OS cell lines, respectively. mCherry (mCherry–C1) and GFP (GFP–C1) expression vectors were transiently transfected into HeLa cells and U2OS cells, respectively, as negative controls. Effects of transient transfection for mCherry–UBF1 (mChe–UBF1), endogenous UBF1 targeted snoMEN without UBF1 expression (UBF1sno), pmCherryUBF1-snoMENv1-PR, non-targeted snoMEN (CM1), endogenous SMN1 targeted snoMEN without GFP–SMN1 expression (SMN1sno) [1], GFP–SMN1, and pGFPSMN1-snoMENv1-PR (SMN1-PR) were also measured. Note, mChe–UBF1 and SMN1sno showed cytotoxic effects when transiently transfected into HeLa cells and U2OS cells, respectively, however, this cytotoxicity was rescued by transfection of UBF1-PR and SMN1-PR. (B) Localisation analysis of endogenous UBF1 (arrow) and mCherry–UBF1 (arrow heads) protein in HeLa cells (HeLa) and HeLa^mCherry–UBF1-PR^ cells (UBF1-PR). Images show localisation pattern of DNA (DAPI, Blue), endogenous UBF1 (TRITC)/mCherry–UBF1 proteins (mCherry, Red), and endogenous RPA194 protein (Green) stained as a Fibrillar Centre (FC) marker [47]. An example of transiently transfecting the mCherry–UBF1 expression plasmid without replacement in HeLa cells is shown in the lower panel (mChe–UBF1). Transiently transfected cells did not show the correct localisation pattern with endogenous UBF1, i.e. FC and nucleoplasm but not Dense Fibrillar Components (DFC) and Granular Components (GC). In addition, these transiently transfected cells show a cytotoxic phenotype (see also Figure 1A). Scale bar is 10 µm. (C) Localisation analysis of endogenous SMN1 (arrow) and GFP–SMN1 (arrow heads) protein in U2OS cells (U2OS), U2OS^GFP–SMN1-PR^ stable cells (SMN1-PR), and U2OS^GFP–SMN1^ stable cells (GFP–SMN1). Images show localisation pattern of DNA (DAPI, Blue), endogenous SMN1 (FITC)/GFP–SMN1 proteins (GFP, Green), and endogenous p80 coilin protein (Red) stained as a Cajal body marker [47]. Scale bar is 10 µm. (D) Expression level of either endogenous UBF1 and mCherry–UBF1 (upper panel), or SMN1 and GFP–SMN1 (lower panel), were measured by western blot analysis. The graphs show average signal intensity and standard deviation for three independent experiments from the same stable clone selected. In the upper panel, the UBF1/mCherry–UBF1 signal ratio was normalised to the tubulin signal. Images on the right side show examples of western blots of UBF1 and SMN1 from established stable cell lines. Ratios were calculated by comparison with endogenous UBF1 and SMN1 signals in control, untransfected cells. An equivalent amount of total cell extract from HeLa cells (HeLa) and HeLa^mCherry–UBF1-PR^ cells (UBF1-PR) was loaded for each lane and the proteins separated by SDS PAGE, electroblotted and probed both with a monoclonal anti-UBF1 antibody and with an anti-tubulin antibody as a loading control. The lower panel shows the same experiment as the upper panel, except SMN1 was detected from U2OS cells (U2OS), U2OS^GFP–SMN1-PR^ stable cells (SMN1-PR), and U2OS^GFP–SMN1^ (GFP–SMN1) stable cells. (E) Gene-expression profiles were compared between U2OS cell and U2OS^GFP–SMN1-PR^ cells by quantitative mass spectrometry analysis. Comparison of expression level of mass spectrometry detected proteins for U2OS cell and U2OS^GFP–SMN1-PR^ cells. Each SILAC experiment was independently repeated at least three times. Correlation between protein ratios of SILAC experiments visualised on a 2D logarithmic graph for all proteins, identified as previously described [48], [49]. On the x and y axis, log_2_ (H/L ratio) correlates with the enrichment in U2OS cells and U2OS^GFP–SMN1-PR^ cells for experiment 1 and experiment 2, respectively. Graph shows a distribution pattern of plot numbers. SILAC ratio values of labelled proteins are listed in Table S1.

The upstream binding factor (UBF) and survival of motor neurons protein (SMN) are examples of proteins where toxic effects of overexpression have been reported. UBF belongs to the sequence-nonspecific class of high mobility group (HMG) proteins and functions in RNA polymerase I transcription [9], [10]. UBF1 depletion using siRNA leads to inhibition of rRNA transcription and cell growth by increasing the number of rRNA genes in an inactive condensed state [9]. UBF1 is a key regulator of cell size and growth, and, therefore, UBF1 overexpression causes strong cytotoxicity [11] (see also Figure 2A). This can explain why it has been problematic to establish a stable cell line that stably expresses FP-tagged UBF1 protein at a reasonable level for both imaging and large-scale biochemical experiments. The survival of motor neurons protein (SMN) is part of a complex involved in the biogenesis of splicing snRNPs (small nuclear ribonucleoproteins). SMN, together with its associated protein complex, has been widely implicated in the assembly of macromolecular complexes essential for splicing and ribosome subunit biogenesis [12], [13], [14]. Previous reports show a lethal phenotype for SMN1 knock-down by siRNA, which induced apoptosis [15]. Also, knock-out of the SMN1 gene in mice is known to be embryonic lethal [16].

In this study, we use the snoMEN-PR vector to establish and characterise human cell lines where expression of either the endogenous UBF1, or SMN, proteins has been reduced and replaced by expression of FP-tagged versions, creating two ‘human protein replacement’ stable cell lines. Furthermore, we investigate the potential mechanism of snoMEN action.

## Materials and Methods

### Cell Culture and Plasmid Construction

HeLa and U2OS cells were provided from EMBL and ATCC, respectively. HeLa and U2OS cells were maintained in Dulbecco’s modified Eagle’s medium (DMEM) supplemented with 10% fetal bovine serum (FBS). All plasmid transfections were performed using effectin (QIAGEN) as described by the supplier.

SnoMEN vectors were established as previously shown [1]. The sequence spanning exon 2 to exon 3 of the C19orf48 gene was inserted 3′ of the EGFP–SMN1/mCherry–UBF1 mammalian expression plasmid (Invitrogen), creating the snoMEN protein replacement vector (Figure 1A). M box sequence of HBII-180C cDNA (5′-CACCCCTGAGGACACAGTGCA-3′) was modified to create complementary sequences to target genes as follows; SMN1∶5′-ATTAGAACCAGAGGCTTGACG-3′, 5′-GCACTGGCTGCGACCTCACCT-3′ and 5′-TTACATTAACCTTTCAACTTT-3′, UBF1∶5′-ACCAACGGTCTGGTAAAGAGT-3′, 5′-GGATCAGTTACCTCATTAGAA-3′ and 5′-CCCACCTTAACTCTCCTCCCC-3′.

### Microscopy and Antibodies

All cell images were recorded using the DeltaVision Spectris fluorescence microscope (Applied Precision). Live cell images for HeLa^mCherry–UBF1-PR^ cells U2OS^GFP–SMN1-PR^ were prepared as previously described (http://www.lamondlab.com/f7protocols.htm). Cells were imaged using a 60× (NA 1.4) Plan Apochromat objective. Twelve optical sections separated by 0.5 µm were recorded for each field and each exposure (SoftWoRx image processing software, Applied Precision). Primary antibodies against UBF1 (F-9, Santa Cruz), SMN1 (BD Transduction Laboratories), RPA194 (C-1, Santa Cruz), tubulin (DM1A, Sigma), coilin (5P10) [17], fibrillarin (72b9) [18], Ago1 (D84G10, Cell Signaling Technology), Ago2 (C34C6, Cell Signaling Technology), and Upf1 (Cell Signaling Technology) were prepared for immunostaining and/or western blotting.

### Proliferation/cytotoxicity Assay

Proliferation/cytotoxicity assay were performed using alamarBlue (AbD serotec) as described by the supplier. Fluorescence was measured using an ELx800 plate reader (BioTek).

### Quantification Analysis of Blotting Images

All signal intensities of blotting images were analysed by imaging software (Image Gauge v4.21; Fujifilm) using manufacturer’s procedure. Briefly, the same size of pixel area was selected and signal intensity calculated by subtraction the background signal. Each signal was normalised with reference to standard control signals, e.g. tubulin, and a signal/control ratio was calculated.

#### SiRNA experiments

SiRNA was transfected by Lipofectamine RNAiMAX (Invitrogen) according to the manufacturer’s instructions. Scrambled siRNA, which does not have an RNA target (Dharmacon), and either SMN1 siRNA (5′-CCAAAUGCAAUGUGAAAUAUU-3′) or UBF1 siRNA (5′-AAAAGUAGCAUUUAAAGACUU-3′) (siMAX siRNA, MWG operon) were transfected as negative and positive controls, respectively. The SMN1 M box siRNAs, targeted to the same intronic sequences as used for the SMN1-PR/UBF1-PR snoMEN, were synthesised and transfected (SMN1 Mbox siRNA-1∶5′-CGUCAAGCCUCUGGUUCUAAU-3′, SMN1 Mbox siRNA-2∶5′-AGGUGAGGUCGCAGCCAGUGC-3′, SMN1 Mbox siRNA-3∶5′-AAAGUUGAAAGGUUAAUGUAA-3′, UBF1 Mbox siRNA-1∶5′-ACUCUUUACCAGACCGUUGGU-3′, UBF1 Mbox siRNA-2∶5′-UUCUAAUGAGGUAACUGAUCC-3′ UBF1 Mbox siRNA-3∶5′-GGGGAGGAGAGUUAAGGUGGG-3′) (siMAX siRNA, MWG operon).

Fibrillarin, Upf1/Rent1, Argonaute-1 (Ago1)/EIF2C1, Argonaute-2 (Ago2)/EIF2C2 siRNAs (On-Targetplus SMART pool product, Dharmacon) were also transfected into U2OS^GFP–SMN1-PR^/U2OS^GFP–SMN1^ stable cell lines using Lipofectamine RNAiMAX.

#### ShRNA experiments

Plasmids to produce shRNAs were constructed using pLVX-shRNA2 vector (Clontech). SMN1 shRNA (5′-AGCGATGATTCTGACATTT-3′)/UBF1 shRNA (5′-CGGAGAAGAAGAAGATGAA-3′) and M box shRNAs were transfected as positive and negative controls, respectively. M box shRNA sequences are as follows; SMN1 Mbox shRNA-1∶5′-GTCAAGCCTCTGGTTCTAA-3′, SMN1 Mbox siRNA-2∶5′-GGTGAGGTCGCAGCCAGTG-3′, SMN1 Mbox siRNA-3∶5′-AAGTTGAAAGGTTAATGTA-3′, UBF1 Mbox siRNA-1∶5′-CTCTTTACCAGACCGTTGG-3′, UBF1 Mbox siRNA-2∶5′-TCTAATGAGGTAACTGATC-3′ UBF1 Mbox siRNA-3∶5′-GGGAGGAGAGTTAAGGTGG-3′.

### Immunoaffinity Purification of FP–SMN1/FP–UBF1 Complexes and Stable Isotope-labelling of Cellular Proteins

SILAC experiments were performed as previously described [19], [20], [21], [22], [23]. Cells were grown for at least six cell divisions in L-arginine-, L-arginine ^13^C_6_^14^N_4_-, or L-arginine ^13^C_6_^15^N_4_-labelling media before analysis. Nuclei were isolated from cells using a variation of a previously described technique (http://www.lamondlab.com/f5nucleolarprotocol.htm). Purified nuclei were resuspended in RIPA buffer to solubilize proteins. Lysates from each cell line were mixed in a 1∶1:1 ratio based on total protein concentration, and FP proteins were affinity purified using anti-GFP/anti-mCherry monoclonal antibodies (GFP–TRAP_A/RFP–TRAP_A, Chromotek).

Isolated nuclear proteins were separated on NuPAGE 4–12% Bis-Tris gel and the gel cut into 12 slices. Peptides resulting from in-gel digestion were extracted from the gel pieces, desalted and concentrated on reverse-phase C18 tips, and eluted into 96-well plates for automated mass spectrometry analysis.

### Mass Spectrometry and Data Analysis

Liquid chromatography-Tandem Mass Spectrometry was performed using an Ultimate U3000 nanoflow system (Dionex Corp) and a linear ion trap-orbitrap hybrid mass spectrometer (LTQ-Orbitrap XL and Velos, Thermo Fisher Scientific Inc.) via a nanoelectrospray ion source (Proxeon Biosystems) as described previously [20]. Data were acquired using Xcalibur software, and quantification was performed using MaxQuant [24], [25] and the Mascot search engine (Matrix Science) for peptide identification against the International Protein Index (IPI) human protein database. The initial mass tolerance was set to 7 p.p.m., and MS/MS mass tolerance was 0.5. Enzyme was set to trypsin/p with up to 3 missed cleavages. Carbamidomethylation of cysteine was searched as a fixed modification, whereas N-acetyl-protein, and oxidation of methionine were searched as variable modifications. A minimum of two peptides were quantified for each protein. An in-house developed software program was used to evaluate peptide identifications and abundance ratios.

## Results

### Establishment of Human Protein Replacement Stable Cell Lines Using snoMEN-PR Vector

**Figure 3:**
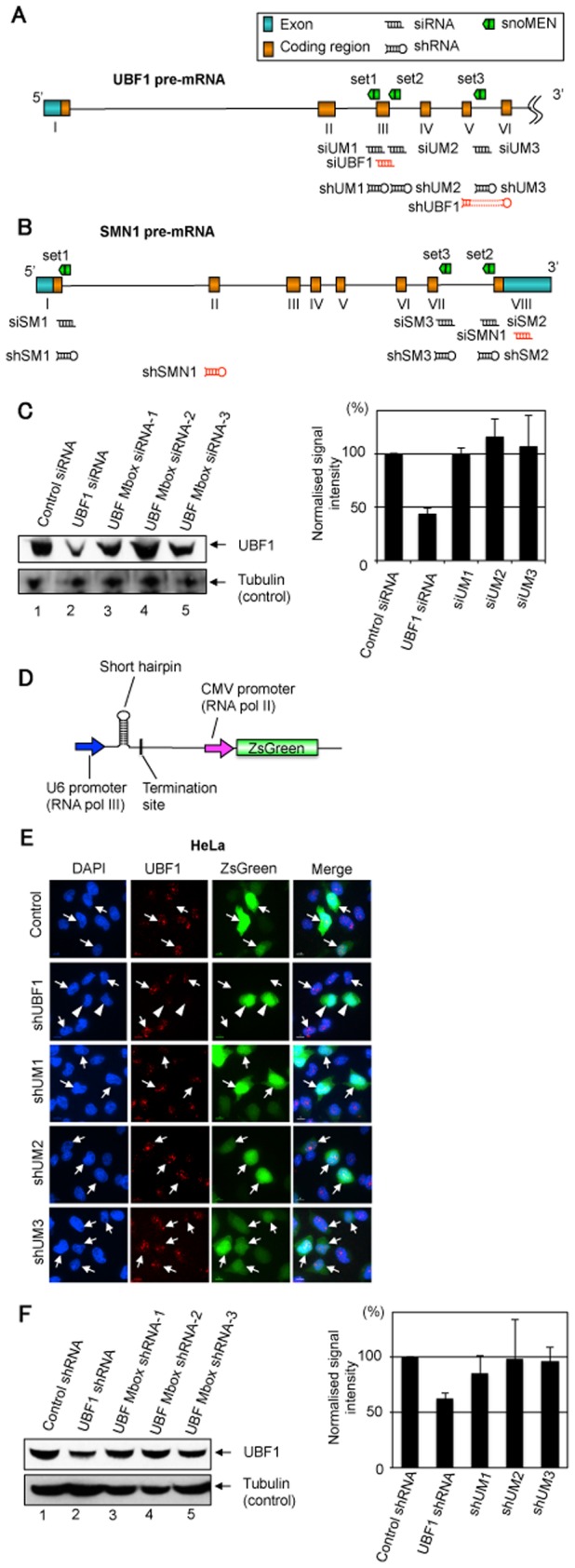
SiRNA and shRNA knock-down targeted to endogenous pre-mRNAs. (A) & (B) The targeted regions on the RNAs for each of the snoMEN vectors used in this study are shown in a schematic diagram. The same pre-mRNA sequence of UBF1 (A)/SMN1 (B) as targeted by the snoMEN vector was targeted by siRNA oligoribonucleotides and shRNA expression plasmids. (C) Western blot analysis for siRNA experiments. Detection of endogenous UBF1 protein levels following transfection of HeLa cells using either Scrambled siRNA (Control: lane1), UBF1 siRNA (siUBF1: lane2), UBF M box siRNA-1 (siUM1: lane3), UBF M box siRNA-2 (siUM2: lane4), and UBF M box siRNA-3 (siUM3: lane5). An equivalent amount of HeLa extract was loaded for each lane and the proteins separated by SDS PAGE, electroblotted onto membrane and probed both with a monoclonal anti-UBF1 antibody and with an anti-tubulin antibody as a loading control. Graph shows UBF1 signal intensity normalised to the tubulin signal measured from three independent experiments. (D) Structure of shRNA expression plasmids. A cDNA producing a short hairpin RNA was subcloned under the U6 RNA polymerase III promoter. Each of the shRNA plasmids encode ZsGreen FP–protein cDNA under CMV promoter regulation as a transfection marker. (E) UBF1 shRNA plasmid and no-endogenous target shRNA plasmid were transfected as a positive and negative control, respectively. UBF1 Mbox shRNA-1 to -3 (shUM1–3) have the same target sequence as UBF1 snoMEN from set1 to set3, respectively (Figure 3A). Scale bar, 10 µm. Arrow: cells not showing knock-down, Arrowhead: cells showing knock-down. (F) Western blot analysis for shRNA experiments. Detection of endogenous UBF1 protein levels following transfection of HeLa cells using either Scrambled shRNA (Control: lane1), UBF1 shRNA (shUBF1: lane2), UBF M box shRNA-1 (shUM1: lane3), UBF M box shRNA-2 (shUM2: lane4), and UBF M box shRNA-3 (shUM3: lane5). An equivalent amount of HeLa extract was loaded for each lane and the proteins separated by SDS PAGE, electroblotted onto membrane and probed both with a monoclonal anti-UBF1 antibody and with an anti-tubulin antibody as a loading control. Graph shows UBF1 signal intensity normalised to the tubulin signal measured from three independent experiments.

Two human protein replacement stable cell lines, HeLa^mCherry–UBF1-PR^ and U2OS^GFP–SMN1-PR^ were established using a procedure previously described [26], [27] except the plasmid used was snoMEN-PR (Figure 1). This resulted in reduced expression of endogenous UBF1 and SMN1 and their replacement with mCherry–UBF1 and GFP–SMN1, respectively. Two vectors expressing M box-modified snoRNAs targeted to endogenous UBF1 and SMN1 pre-mRNAs were constructed, based on the previous snoMEN design [1]. Plasmids pmCherry–UBF1snoMENv1-PR and pGFP–SMN1snoMENv1-PR encode mCherry–UBF1 and GFP–SMN1 cDNA, respectively, for the knock-in of the FP-tagged UBF1 and SMN1, and three M box-modified snoRNAs, each targeted to different exon–intron junction positions within endogenous UBF1 and SMN1 pre-mRNAs, for the knock-down of expression of the endogenous gene (Figure 1A) (see also methods, Figure 3A and B). pmCherry–UBF1snoMENv1-PR and pGFP–SMN1snoMENv1-PR, which also contain the neomycin-resistance gene, were transfected into HeLa and U2OS cells, respectively (Figure 1B). Twenty four hours after transfection, G418 was added into the culture medium for selection of transfected cells. After two weeks of G418 selection, multiple positive clones were independently chosen and assigned as passage 1 (p1) and cultured for more than 14 passages before analysis to confirm stable expression of FP-tagged proteins (Figure 1B). Both stable cell lines, HeLa^mCherry–UBF1-PR^ and U2OS^GFP–SMN1-PR^, show expression of FP-tagged proteins at levels sufficient for imaging by fluorescence microscopy (Figure 1C and see also Figure S1A).

### Characterisation of snoMEN-PR Stable Cell Lines

The growth rate of each stable cell line was examined by a proliferation assay (Figure 2A). Both HeLa^mCherry–UBF1-PR^ [left panel, UBF1-PR (stable)] and U2OS^GFP–SMN1-PR^ [right panel, SMN1-PR (stable)] cell lines show similar growth rates compared with host cells, i.e. HeLa and U2OS cells, respectively. Transient transfection of mCherry–C1, which expresses mCherry protein alone, and pmCherry–UBF1snoMENv1-PR (UBF1-PR), did not cause either cytotoxicity, or growth suppression. However, transient transfection of mCherry–UBF1 expression plasmid (mChe–UBF1) and UBF1 snoMEN expression plasmid (UBF1sno) resulted in both cytotoxicity and growth suppression, consistent with previous studies [9], [11] (Figure 2A left panel, see also Figure 2B mChe–UBF1). These results suggested that the previous problems of cytotoxicity/growth-suppression upon UBF1 overexpression/depletion were circumvented with snoMEN protein replacement. Thus, even although the level of protein replacement is not complete, it is sufficient to rescue cell viability. The growth rate of the HeLa^mCherry–UBF1-PR^ stable cell line, which stably expresses mCherry–UBF1 and constitutively knocks down endogenous UBF1 expression, is normal.

Transient transfection of GFP–C1 (expressing GFP protein alone), CM1 (which expresses snoMEN with no endogenous target [1]), GFP-SMN1, and pGFP–SMN1snoMENv1-PR (SMN1-PR) did not cause either cytotoxicity, or growth suppression. However, transient transfection of the SMN1 snoMEN expression plasmid (SMN1sno) resulted in cytotoxicity, consistent with previous studies [1], [15], [26] (Figure 2A right panel). These results suggested that the cytotoxicity caused by SMN1 depletion was rescued by snoMEN protein replacement and that the growth rate of the U2OS^GFP–SMN1-PR^ stable cell line, which stably expresses GFP–SMN1 and constitutively reduces endogenous SMN1 expression, was normal.

The localisation patterns of the tagged proteins in the HeLa^mCherry–UBF1-PR^ and U2OS^GFP–SMN1-PR^ stable cell lines were examined by immunostaining. In both stable cell lines, the tagged UBF1 and SMN1 proteins co-localise with nucleolar and Cajal body marker proteins, i.e. RPA194 and coilin, respectively, consistent with the localisation pattern of the endogenous proteins (Figure 2B and C, arrowhead and arrow). In the case of mCherry–UBF1 overexpression, the majority of the tagged protein shows no co-localisation with RPA194 in nucleoli (Figure 2B mChe–UBF1) and these transfected cells show a cytotoxic phenotype after 24 hours.

Western blot analysis was performed to measure the protein replacement ratio for HeLa^mCherry–UBF1-PR^ and U2OS^GFP–SMN1-PR^ stable cell lines (Figure 2D). Both cell lines show about 40% reduction of the levels of endogenous UBF1/SMN1 proteins and 30–40% knock-in of FP-tagged proteins (Figure 2D, graphs UBF1-PR and SMN1-PR). It was not possible to generate a stable cell line for the overexpression of mCherry–UBF1 that also stably expressed the FP-tagged protein, which is consistent with the results of the proliferation assay (Figure 2A) and also as reported in a previous study [11]. To establish protein replacement stable cell lines, clones were selected where the FP-tagged protein was expressed only at a level comparable to the level of knock-down of the endogenous protein, thereby avoiding net overexpression.

The expression profile of a wide range of genes in both U2OS^GFP–SMN1-PR^ and HeLa^mCherry–UBF1-PR^ stable cell lines was compared using SILAC (Stable Isotope Labelling of Amino acids in Cell culture) quantitative mass spectrometry (MS) analysis [19], [20], [21], [22], [28] (Figure 2E and Figure S1B). From the approximately 700 proteins detected by MS, more than 680 proteins (97.4%) showed less than a two-fold difference in expression between host cells and snoMEN-PR stable cells. In combination, these results demonstrate the establishment of human protein replacement stable cell lines, including a cell line previously not technically possible, using the snoMEN-PR vector.

### Comparison of siRNA/shRNA and snoMEN RNA Interference

The data above show that M box-modified snoRNAs can reduce expression of endogenous cell proteins when targeted to sequences within introns of pre-mRNAs and intron/exon junction sequences that are not present in the mature mRNA. We compared this with the ability of siRNA oligoribonucleotides to knock-down expression of both the UBF1 and SMN1 proteins when targeted against the same intronic sequences (Figure 3 and Figure S2). Therefore, for both UBF1 and SMN1, three siRNA oligonucleotides per gene complementary to the same exon-intron/intron sequences in either UBF1, or SMN1, pre-mRNAs as targeted by the M box-modified snoRNAs in pmCherry–UBF1snoMENv1-PR and pGFP–SMN1snoMENv1-PR, were transfected into HeLa and U2OS cells (Figure 3A and B). All three intron-targeted siRNAs (siUM1–3) showed little or no knock-down of UBF1, as did a further negative control siRNA, as judged by protein blotting (Figure 3C) and by immunofluorescence (data not shown). As a positive control, another siRNA targeted to UBF1 exon 3 sequence (siUBF1), resulted in ∼50% knock-down. A similar result was obtained using three siRNAs targeted to introns 1 & 7 of SMN1 (siSM1–3), which also failed to knock-down, as did a further negative control siRNA, while a positive control siRNA targeted to SMN1 exon 8 reduced SMN1 levels by ∼30% as judged by both immunofluorescence and protein blotting analyses (Figure S2A and B). Furthermore, the same series of analyses were performed using shRNA technology (Figure 3A, B, D, E and F Figure S2C and D). Three shRNA expression plasmids per gene, complementary to the same exon–intron/intron sequences in either UBF1, or SMN1 pre-mRNAs, as targeted by the M box-modified snoRNAs in pmCherry–UBF1snoMENv1-PR and pGFP–SMN1snoMENv1-PR, were transfected into HeLa and U2OS cells (Figure 3A, B, and D). All three intron-targeted shRNAs (shUM1–3) showed little or no knock-down of UBF1, compared with non-transfected cells (Figure 3E and F). As a positive control, another shRNA plasmid targeted to a UBF1 exon 5 & 6 sequence (shUBF1), resulted in ∼30% knock-down. A similar result was obtained using three shRNA expression plasmids targeted to the intron of SMN1, which showed little or no knock-down of expression compared with non-transfected cells, while a positive control shRNA plasmid targeted to SMN1 exon 2 sequence reduced SMN1 levels by ∼70% (Figure S2C and D).

We conclude that the snoRNA vectors can target RNA sequences to reduce expression of genes, including targets that are not amenable to knock-down by both siRNA and shRNA mechanisms. The combined results indicate that the mechanism of knock-down is dependent on snoRNA expression, that snoRNAs are nuclear and that they can knock-down target intron RNA sequences that are not present in cytoplasmic mRNA.

**Figure 4:**
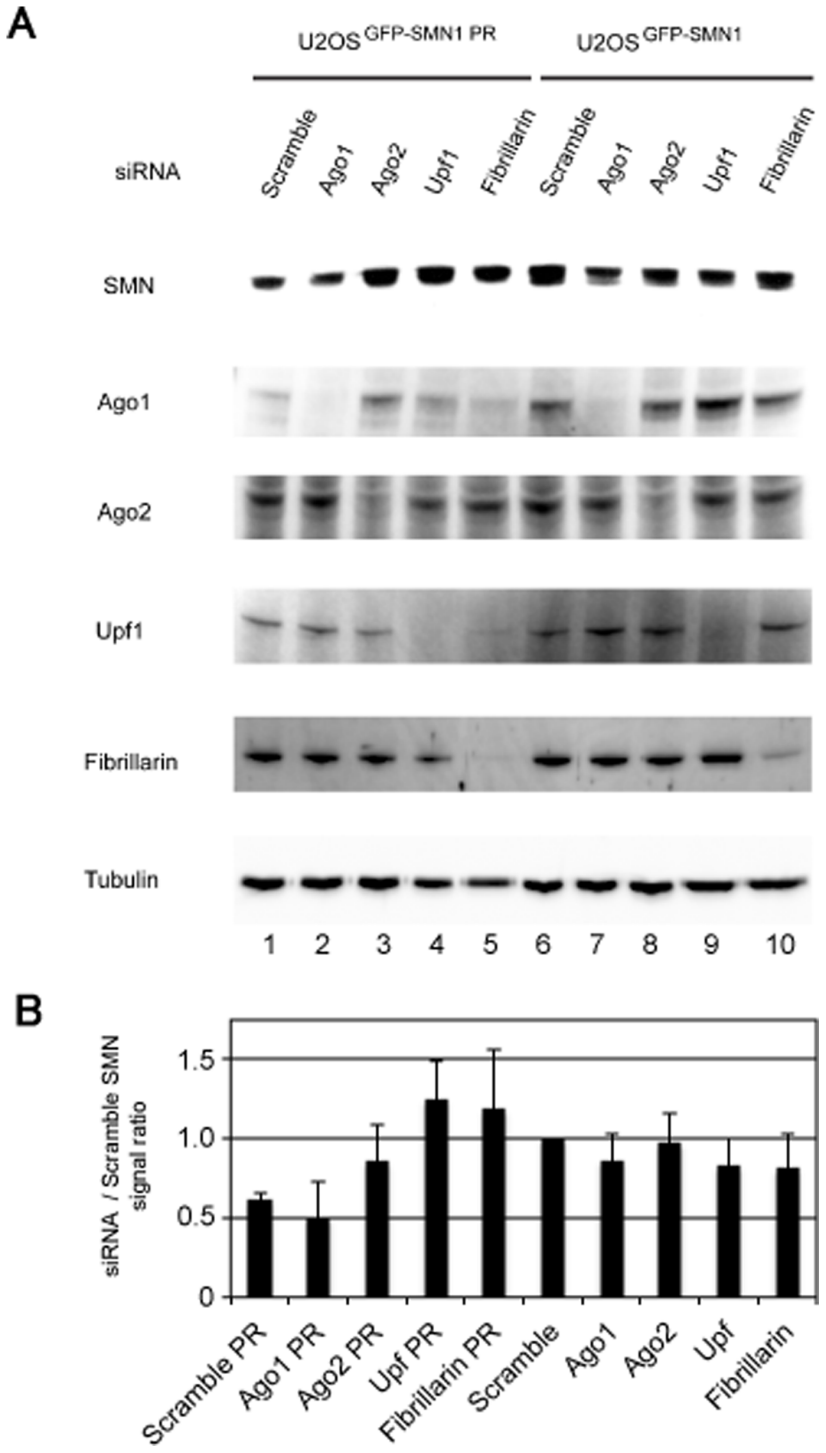
Potential factors involved in snoMEN machinery. (**A**) U2OS^GFP–SMN1-PR^ and U2OS^GFP–SMN1^ cells were transfected with either Scrambled siRNA, Ago1 siRNA, Ago2 siRNA, Upf1 siRNA, or fibrillarin siRNA. An equivalent amount of each extract was loaded in each lane and the proteins were separated by SDS PAGE, electroblotted onto membrane and probed with anti-SMN1, anti-Ago1, anti-Ago2, anti-Upf1, anti-fibrillarin and with anti-tubulin as a loading control. (**B**) The graphs show average SMN signal intensity and standard deviation for three independent experiments using the same procedure as in A. SMN signal ratio was normalised to the tubulin signal.

Little is known about the molecular mechanism of gene regulation by snoRNAs. However, some snoRNAs have been reported to associate with Argonaute proteins (Ago1, or Ago2), which are involved in the main RNAi pathway [29]. Therefore, we examined whether RNAi-mediated depletion of several proteins, including Ago2, could affect the snoMEN machinery (Figure 4). This result showed that the gene suppression effect of the snoMEN against SMN was reduced in either Ago2, up-frameshift-1 (Upf1), or fibrillarin depleted cells, compared to the control in lane 1 (Figure 4). This implies that these proteins may be involved, either directly or indirectly, in the mechanism of snoMEN-mediated gene suppression. Interestingly, depletion of Upf1, which is thought to be essential to nonsense-mediated decay (NMD) [30], [31], [32], also prevented the inhibition of gene expression by snoMEN. We have previously shown that both box C and box D motifs are required for snoMEN expression [1]. Fibrillarin binds endogenous snoRNAs via a box C-D base paired structure, termed the k-turn, and is necessary for snoRNA maturation and expression. Therefore, depletion of fibrillarin might decrease snoMEN expression levels and thereby prevent the gene suppression effect (Figure S3). Taken together, our results suggest that snoMEN might modulate target gene expression via a mechanism involving Ago2 and/or Upf1.

### MS Pull-down Assay of FP-proteins Using snoMEN-PR Stable Cell Lines

**Figure 5:**
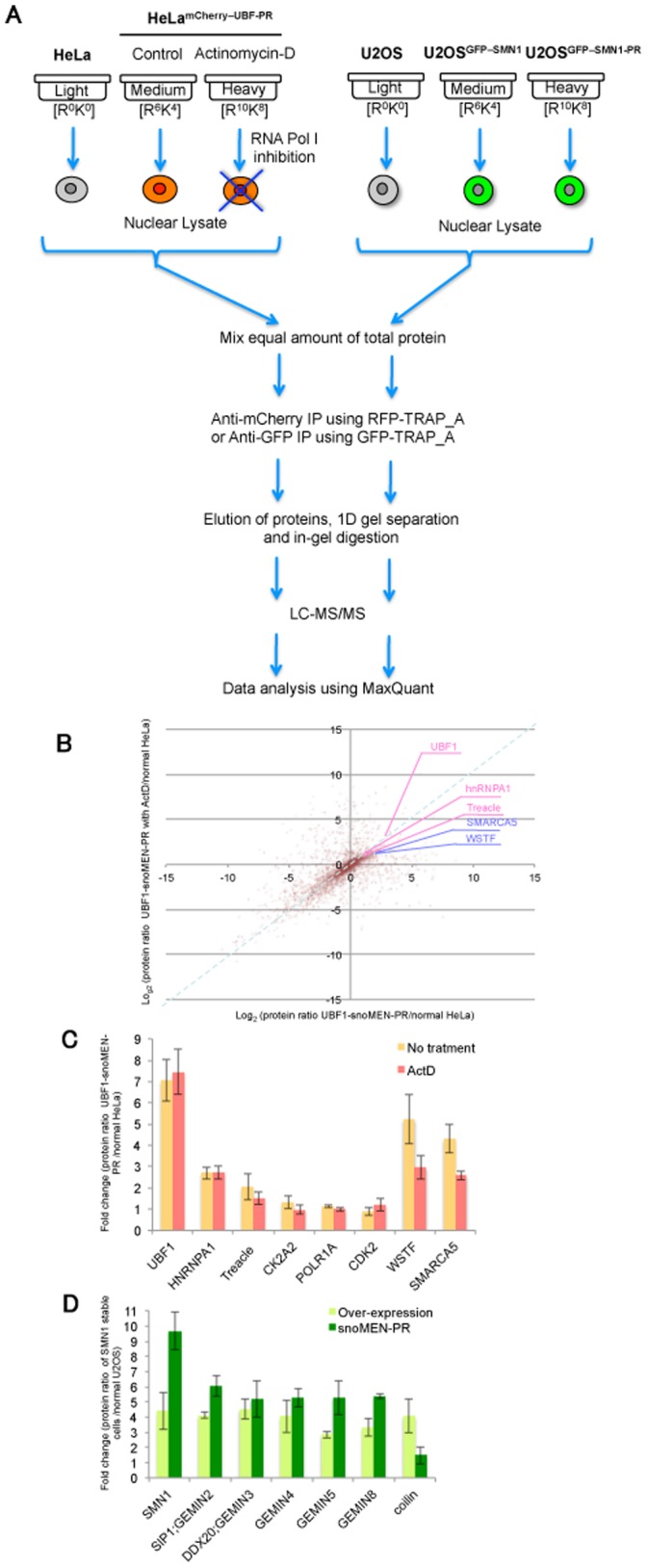
Characterisation of FP–protein complexes in replacement stable cell lines by Quantitative SILAC Proteomic analysis. (**A**) Design of the triple-encoding SILAC pull down experiments (see text). Comparison of mCherry–UBF1 complex, either in the presence, or absence, of low concentration Actinomycin-D (left panel) and comparison of GFP–SMN1 complex either with replacement (U2OS^GFP–SMN1-PR^), or without replacement (U2OS^GFP–SMN1^) (right panel). The SILAC experiments were independently repeated at least four times. (**B**) SILAC result of mCherry–UBF1 complex pull down assay visualised on a 2D logarithmic graph for all proteins identified. On the x axis, log_2_ (M/L ratio) correlates with the enrichment in mCherry–UBF1 IP versus control IP. On the y axis, log_2_ (H/L ratio) correlates with the enrichment in mCherry–UBF1 IP with Actinomycin-D treatment versus no-treatment IP. The bait, UBF1, is shown in red, and a blue line separates the proteins whose interaction with UBF1 is increased (above the line)/or decreased (below) after Actinomycin-D treatment. Known interaction partners, which were identified and quantified, are also highlighted. SILAC ratio values of labelled proteins are listed in Table S2. (**C**) Known UBF1 interaction partners, which were identified and quantified. Graph shows fold change of each protein ratio with Actinomycin-D treatment (red) and without treatment (orange) measured from five independent experiments. (**D**) Comparisons of protein ratios of known interaction partners of SMN1 between over expression stable cell line (U2OS^GFP–SMN1^; light green) and protein replacement stable cell line (U2OS^GFP–SMN1-PR^; green) measured from five independent experiments.

The establishment of snoMEN-PR stable cell lines led us to examine how they may be used for large-scale biochemical experiments, such as pull-down assays. Two series of quantitative, MS-based SILAC proteomics experiments were used to characterise a) changes in UBF1 complexes in either the absence, or presence, of low concentrations of Actinomycin-D, i.e. comparing when RNA polymerase I is either active, or inactive, respectively (Figure 5A left panel, and see also Figure S4), b) differences in the incorporation ratio into *in vivo* SMN1 complexes between over expression (U2OS^GFP–SMN1^) and protein replacement stable cells (U2OS^GFP–SMN1-PR^) (Figure 5A, right panel). HeLa^mCherry–UBF1-PR^ and a triple isotope SILAC labelling scheme was employed [19]. Control HeLa/U2OS cells were cultivated in medium containing the normal, “light” amino acids, while cells stably expressing mCherry–UBF1/GFP–SMN1 were grown either with “medium” (no treatment/without endogenous SMN1 replacement), or with “heavy” (Actinomycin-D treatment/with endogenous SMN1 replacement), isotope-labelled amino acids. The conditions of Actinomycin-D treatment were carefully titrated (Figure S4) [28]. Soluble cell extract was prepared from each of the light, medium, and heavy cell cultures; mCherry–UBF1/GFP–SMN1 and associated partners were affinity purified and tryptic peptides were analysed by MS. Intensity ratios for the three isotopic forms of each protein were determined using MaxQuant [24], [25]and analysed. Data were plotted in 2D logarithmic graphs separately for UBF1 (Figure 5B) and SMN1 (Figure S5A).

The result of the UBF1 pull-down is shown in Figure 5B: the x axis represents enrichment of mCherry–UBF1-associated proteins in comparison with the control IP (medium/light ratio) and the y axis represents enrichment of mCherry–UBF1-associated proteins in Actinomycin-D-treated versus untreated cells (heavy/light ratio). Contaminant proteins are clustered around the origin, while proteins specifically co-purifying with mCherry–UBF1 in untreated cells appear on the upper right of the graph. Proteins whose specific co-purification with mCherry–UBF1 decreased upon treatment with Actinomycin-D appear below the 1∶1 line (Figure 5B, dashed blue line). The UBF1 ratio is 1∶1, as expected, which means that the same amount of UBF1 protein was pulled down in both conditions (i.e. with and without Actinomycin-D treatment). Many of the UBF1 directly/indirectly associated proteins that were quantified, e.g. hnRNPA1, TCOF1 (Treacle), CK2A2 (Casein kinase II, alpha 1), POLR1A (polymerase (RNA) I polypeptide A), and CDK2 (Cyclin dependent kinase 2) [33], [34], [35], [36], also showed little or no change in ratio, similar to UBF1. However, the ratio of other proteins, especially those associated with chromatin remodelling, e.g. SMARCA5/SNF2h (SWI/SNF related, matrix associated, actin dependent regulator of chromatin, subfamily a, member 5), WSTF (Williams syndrome transcription factor) 37, was decreased by Actinomycin-D treatment (Figure 5C).

In Figure 5D and Figure S5, displaying the data from the GFP–SMN1 pull-down assay, the x axis represents enrichment of GFP–SMN1-associated proteins (protein replacement cell line IP versus the control IP: heavy/light ratio) and the y axis represents enrichment of GFP–SMN1-associated proteins (over-expression cell line IP versus the control IP: medium/light ratio) (Figure S5). Contaminant proteins are clustered around the origin, while proteins specifically co-purifying with GFP–SMN1 in over expressed cells appear to the bottom right of the graph. Proteins whose specific co-purification with GFP–SMN1 increased in protein replacement, compared with over expression, appear above the 1∶1 line (Figure S5A and B, dashed red line). Interestingly, the pull-down ratio of SMN1 in the replacement cell line shows a more than 2-fold increase compared with the over-expression cell line, even though GFP–SMN1 expression in the replacement cell line is less than 40% of the endogenous protein level (Figure 2D, Figure 5D and Figure S5B). As well as SMN1, the majority of the previously identified nuclear SMN1 interaction partners, e.g. Gemin family proteins (Gemin2, 5, and 8), also show an increased SILAC ratio in the replacement cell line compared with the overexpression cell line (Figure 5D and Figure S5B). It is known that SMN1 forms an octamer complex both *in vivo* and *in vitro* [38]. These results suggest that FP-tagged SMN1 protein is likely incorporated into endogenous complexes more efficiently in the SMN1 replacement cell line, U2OS^GFP–SMN1-PR^, than in a typical overexpression stable cell line, U2OS^GFP–SMN1^ (see also a model in Figure S5C).

## Discussion

**Figure 6:**
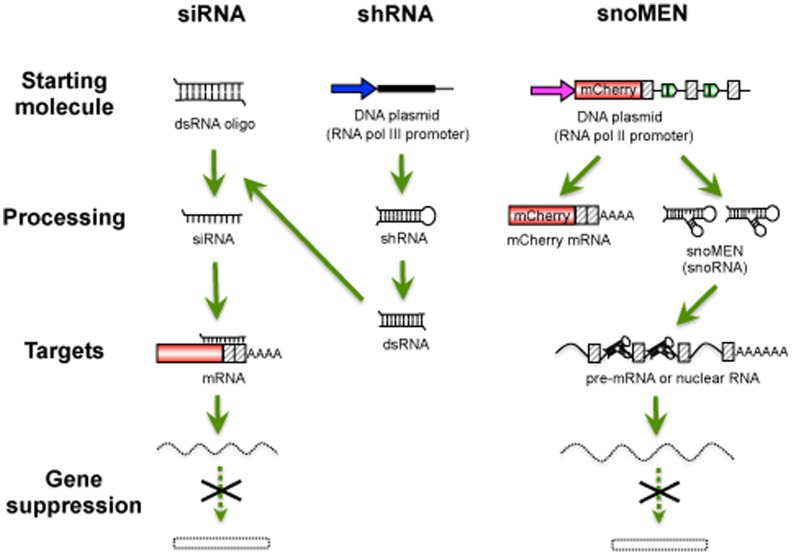
Features of snoMEN technology. Schematic diagram showing differences between the siRNA/shRNA and snoMEN systems. Arrows show promoters for RNA polymerase III (shRNA) and RNA polymerase II (snoMEN), respectively. Red squares show the coding region, e.g. either mCherry cDNA, or endogenous genes. Striped squares show non-coding exon region. The bars show non-coding regions, e.g. introns.

In this study we describe the establishment of human protein replacement stable cell lines using the snoMEN vector technology (snoMEN-PR) and analyse these snoMEN-PR stable cell lines using fluorescence microscopy and quantitative mass spectrometry. The snoMEN vector was derived from a human box C/D snoRNA (HBII-180C). We demonstrated previously that the modified HBII-180C snoRNA backbone, i.e. snoMEN, could modulate targeted gene expression for both cellular genes and G/YFP-fusion proteins, using transient transfection methods [1]. Gene knock-down is mediated via a short internal snoRNA region, termed the M box, which can be manipulated to make it complementary to a target RNA sequence of choice. Briefly, the major differences between snoMEN technology and other knock-down systems are a) snoMEN target nuclear RNAs, e.g. pre-mRNAs and non-coding RNAs, b) snoMEN RNAs are transcribed from RNA polymerase II promoters instead of the RNA polymerase III promoter required for shRNA plasmids, c) multiple snoMEN RNAs can be incorporated within a single transcript under the regulation of a single promoter [1] (Figure 6).

We show here for two separate proteins, i.e., UBF and SMN, that a subset of the total pool of endogenous protein can be knocked down and that fraction replaced by an exogenous, tagged version of the same protein, that is expressed from the same transcript in the same vector that is used for the knock-down. Even although the efficiency of replacement here is partial, it is nonetheless demonstrated to be sufficient to allow the establishment of stable cell lines for proteins that are toxic when simply overexpressed in the presence of unaltered levels of the endogenous factors. This demonstrates the value of the approach, which is further illustrated by the observation, based upon immunoprecipitation experiments, that there is more efficient incorporation of the tagged, exogenous version of proteins into multiprotein complexes in the snoMEN-PR cell lines as compared with cells overexpressing the tagged protein in competition with the endogenous version. It should be noted that it is always to be expected that knock-down strategies will in practice result in incomplete removal of the targeted protein and that only chromosomal deletion or mutation of the cognate gene will guarantee complete removal of the protein. We anticipate that the efficiency of protein replacement using the snoMEN strategy can be enhanced in future by further improvements in the vector design but the present study already illustrates valuable applications of the current technology.

In this study the data suggest that snoMEN might modulate target gene expression via a mechanism involving Ago2 and/or Upf1 (Figure 4). Although both Ago2 and Upf1 localise mainly in the cytoplasm, where they function for RNA interference [6], [7], [8], [39] and NMD [30], [31], [32] respectively, analysis of the spatial proteome of human cells show that a proportion (∼30–40%) of both Ago2 and Upf1 proteins may also localise in the nucleus [40], [41] [analysed within Peptracker ( [42])]. In addition, reports of nuclear functions for Ago2 and Upf1 are currently expanding [43], [44], [45]. It might be possible that snoMEN RNA interference occurred by an Ago2 dependent RNA cleavage event and/or by blocking splicing of target mRNA involved in the Upf1 NMD pathway. Further study is necessary to define how Ago2 and/or Upf1 may be involved in the snoMEN mechanism of action.

Characterisation of snoMEN-PR stable cell lines showed that stable snoMEN expression does not prevent or reduce cell growth, or alter the expression profile of a wide range of genes, as measured by a quantitative MS approach (Figure 2E and Figure S1B). The relatively high dose of siRNA often required for high efficiency gene silencing may saturate endogenous RNAi pathways leading to indirect effects on cell growth and gene expression [6]. However, the snoMEN system benefits from the fact that endogenous box C/D snoRNAs are highly abundant nuclear RNAs that are efficiently processed from within introns of many different protein-coding cellular pre-mRNAs. M box-modified snoRNAs are thus processed efficiently from vector transcripts with reduced chance of overloading the endogenous snoRNA processing machinery, which is consistent with the gene-profile analysis in this study (Figure 2E and Figure S1B).

Establishment of UBF1 protein replacement stable cell lines, HeLa^mCherry–UBF1-PR^, allowed us to perform large-scale biochemical experiments, e.g. pull-down analysis, which were previously difficult for the reasons outlined above. We identified both known and novel potential interaction partners of UBF1, either with, or without, RNA polymerase I inhibition, i.e. Actinomicyn-D treatment (Figure 5B, C). For example, known interaction partners, e.g. RNA polymerase I (Pol I) subunits, showed no change after Pol I inhibitiessenger RNA surveillance: neutralizing natural nonseon; however, the interaction of UBF1 with some of the chromcomplex targets heterochromatic replication foci.atin remodelling associated proteins, e.g. SMARCA5/SNF2h, and WSTF, was decreased by Actinomycin-D treatment (Figure 5C). WSTF and SNF2h are components of a chromatin remodelling complex, termed WICH, that is recruited to replication foci and prevents aberrant heterochromatin formation shortly after DNA replication, thereby allowing rebinding of factors to newly replicated DNA [46]. The complex colocalises and interacts with RNA Pol I in nucleoli, and RNA interference-mediated knock-down of WSTF impairs pre-rRNA synthesis. Previous results indicate that a 23 MDa multiprotein complex containing WSTF and SNF2h is required for rDNA transcription [37]. These results, combined with our quantitative pull-down assay of FP-tagged UBF1 protein, suggest that RNA polymerase I inhibition by Actinomycin-D treatment disrupts the interactions between UBF1 and chromatin remodelling complexes, but not the interaction of UBF1 with RNA polymerase I subunits. Further study is needed to reveal the detailed mechanism by which the RNA polymerase I machinery and UBF1-chromatin remodelling complex interact. Furthermore, the SILAC pull-down assay demonstrated an improved recovery of interacting proteins using the protein replacement (U2OS^GFP–SMN1-PR^) stable cell line, compared with the over-expression stable cell line, U2OS^GFP–SMN1^ (Figure 5D and Figure S5). This occurs even though FP-tagged SMN1 protein expression levels are almost 40% lower in U2OS^GFP–SMN1-PR^ than U2OS^GFP–SMN1^ cells (Figure 2D). It is known that SMN1 forms an octamer complex both *in vivo* and *in vitro* [38]. These results suggest that FP-tagged SMN1 is more efficiently incorporated into the endogenous octamer complex in the snoMEN protein replacement stable cell line, likely because this system does not have to force the incorporation of FP-tagged proteins into endogenous complexes (see model in Figure S5C). Interestingly, a few proteins, e.g. coilin (Figure 5D), show the opposite result, i.e. better recovery with the increased SMN1 expression levels as seen in the over-expression stable cell line, U2OS^GFP–SMN1^. This may suggest that the proteins in this category, such as coilin, mainly bind to free SMN1, but not to the SMN1 complex. This would be an interesting point for future investigation.

The snoMEN vectors provide an alternative tool for studying gene expression that is complementary to existing methods. The ability to quickly replace a significant proportion of essential endogenous proteins with tagged and/or mutant versions in stable cell lines is likely to prove particularly useful. For example, the snoMEN approach could potentially be useful to express catalytically-deficient versions of enzymes, such as methyl transferases, and endoRNases. We anticipate that further improvements in snoMEN vectors design may increase further the proportion of endogenous protein that can be replaced and we foresee future applications for snoMEN vectors in basic gene-expression research, in drug screening and target validation studies and possibly also for gene therapy. All of these applications can benefit from the ability to deliver knock-down and protein replacement RNAs from a single vector encoding a single transcript. The snoMEN vectors thus expand the repertoire of technologies available for manipulating gene expression in mammalian cells and can provide new opportunities for overcoming current limitations. Furthermore, we also foresee a development of snoMEN vectors to expand their utility and applications, e.g. incorporating inducible promoters and delivery using virus based vectors.

## Supporting Information

### Supplementary Materials and Methods

#### *In vivo* transcription assay

*In vivo* transcription assays were performed as previously described (http://www.lamondlab.com/f7protocols.htm). 1 mM of 5-fluorouridine (FU) was added into the culture medium 15 min before fixation. Incorporated FU was detected by staining with an Anti-BrU antibody (B2531, Sigma).

Figure S1 Gene-expression profile of snoMEN replacement stable cell lines. (A) Distribution pattern of GFP-SMN1 signal intensity of U2OS^GFP–SMN1-PR^ stable cell line. Cytoplasmic GFP signals were calculated from randomly selected cells (n = 42). Each signal was normalised by DAPI signal. (B) Expression level comparison of proteins detected by Mass spectrometry for HeLa^mCherry–UBF1-PR^ versus HeLa cells. Each SILAC experiment was independently repeated at least three times. Correlation between protein ratios of SILAC experiments visualised on a 2D logarithmic graph for all proteins identified as previously demonstrated [48], [49]. On the x and y axis, log_2_ (H/L ratio) correlates with the enrichment in HeLa^mCherry–UBF1-PR^ versus HeLa cells for experiment 1 and experiment 2, respectively. Graph shows a distribution pattern of plot numbers. SILAC ratio values of labelled proteins are listed in Table S3.
(TIF)

Figure S2 SiRNA and shRNA knock-down targeted to endogenous SMN1 pre-mRNAs. (A–D) These are the same experiment as in Figure 3C–F except the target gene is SMN1 in U2OS cells. (A) Scrambled siRNA (Negative control siRNA) and SMN1 siRNA (Dharmacon) were transfected as a negative and a positive control, respectively. SMN1 Mbox siRNA-1 to -3 (siSM1–3) have the same target sequence as SMN1 snoMEN from set1 to set3, respectively (Figure 3A). Scale bar, 10 µm. Arrowhead: cells showing knock-down. (B) Western blot analysis for siRNA experiments. Detection of protein levels for endogenous SMN1 following transfection of U2OS cells using either Scrambled siRNA (Control: lane1), SMN1 siRNA (siSMN1: lane2), SMN M box siRNA-1 (siSM1: lane3), SMN M box siRNA-2 (siSM2: lane4), and SMN M box siRNA-3 (siSM3: lane5). An equivalent amount of U2OS extract was loaded for each lane and the proteins separated by SDS PAGE, electroblotted onto membrane and probed both with a monoclonal anti-SMN1 antibody and with anti-tubulin as a loading control. Graph shows SMN1 signal intensity normalised to the tubulin signal measured from three independent experiments. (C) A shRNA plasmid targeted to SMN1 and no-endogenous target shRNA plasmid were transfected as a positive and negative control, respectively. SMN1 Mbox shRNA-1 to -3 (shSM1–3) have the same target sequence as SMN1 snoMEN from set1 to set3, respectively (Figure 3B). Scale bar, 10 µm. Arrow: cells not showing knock-down, Arrowhead: cells showing knock-down. (D) Western blot analysis for shRNA experiments. Detection of protein levels for endogenous SMN1 following transfection of U2OS cells using either Scrambled shRNA (Control: lane1), SMN1 shRNA (shSMN1: lane2), SMN M box shRNA-1 (shSM1: lane3), SMN M box shRNA-2 (shSM2: lane4), and SMN M box shRNA-3 (shSM3: lane5). An equivalent amount of U2OS extract was loaded for each lane and the proteins separated by SDS PAGE, electroblotted onto membrane and probed both with a monoclonal anti-SMN1 antibody and with anti-tubulin as a loading control. Graph shows SMN1 signal intensity normalised to the tubulin signal measured from three independent experiments.
(TIF)

Figure S3 SnoRNA expression analysis after Fibrillarin knock-down treatment. qRT-PCR was performed to measure snoRNA expression level after treatment with scramble siRNA (Control) and Fibrillarin siRNA. Equal amounts of total RNA from U2OS^GFP–SMN1-PR^ cells, extracted following siRNA treatment, was used for qRT-PCR reactions. Graph shows the snoRNA expression ratio between control and fibrillarin siRNA experiments measured from four independent experiments. SnoRNA HBII-180C (snoMEN backbone) specific primers and GAPDH mRNA specific primers, as a loading control, were used for amplification.
(TIF)

Figure S4 Optimisation of RNA polymerase I inhibition using low concentration Actinomycin-D treatment. (A) *In vivo* transcription assay with/without Actinomycin-D treatment in HeLa cells. HeLa cells were treated either with ethanol (EtOH) as a negative control, or with Actinomycin-D (0.01 µg/ml) for each time point: 30 min, 1 hr, and 2 hr. Transcription in the cells was detected via incorporation of 5-fluorouridine. Two hours following Actinomycin-D treatment, the nucleolar signal had disappeared (arrow). Scale bar indicates 14 µm. (B) Identification of pre-rRNA transcriptions. Northern blot analysis was performed to decide a time point of pre-rRNA inhibition by Actinomycin-D. Each pre-rRNA was detected by using probes specific to 5.8S, 18S, and 28S rRNAs. U3 snoRNA was also detected as a loading control. (C) Specific RNA polymerase I inhibition was confirmed by imaging fibrillarin and coilin localisation patterns. Fibrillarin accumulated only in nucleoli after low concentration Actinomycin-D treatment for 2 hr (arrow); however, the accumulation of coilin that should occur at the nucleolar cap on inhibition of RNA polymerases I, II and III with high concentration Actinomycin-D treatment (1 µg/ml) [28], was not seen.
(TIF)

Figure S5 Characterisation of FP–protein complexes in replacement stable cell lines by Quantitative SILAC Proteomic analysis. (**A**) SILAC result of GFP–SMN1 complex pull-down assay visualised on a 2D logarithmic graph. On the x axis, log_2_ (H/L ratio) correlates with the enrichment in GFP–SMN1 IP with protein replacement versus control IP. On the y axis, log_2_ (M/L ratio) correlates with the enrichment in GFP–SMN1 IP versus control IP without replacement. The bait, SMN1, is highlighted in green, and a red line separates the proteins whose interaction with SMN1 is increased (above the line), or decreased (below) by protein replacement. SMN1 binding proteins which were identified and quantified are highlighted in B. SILAC ratio values of labelled proteins are listed in Table S4. (**B**) The graph [expanded top right segment of (a)] shows SILAC fold change ratio for known SMN binding proteins in the GFP–SMN1 IP. (**C**) The model of immuno–precipitation of endogenous SMN1 complex.
(TIF)

Table S1 **The list of SILAC ratio values of labelled proteins described in** **Figure 2E** **.**
(XLSX)

Table S2 **The list of SILAC ratio values of top 10% labelled proteins described in** **Figure 5B** **.**
(XLSX)

Table S3 **The list of SILAC ratio values of labelled proteins described in Figure S1B.**
(XLSX)

Table S4 **The list of SILAC ratio values of top 10% labelled proteins described in Figure S5A.**
(XLSX)
