# Supplementary figures and images for: Analysis of Human Protein Replacement Stable Cell Lines Established using snoMEN-PR Vector
Source: PLoS One. 2013 Apr 25;8(4):e62305. doi: 10.1371/journal.pone.0062305 (PMC3636044; doi:10.1371/journal.pone.0062305)

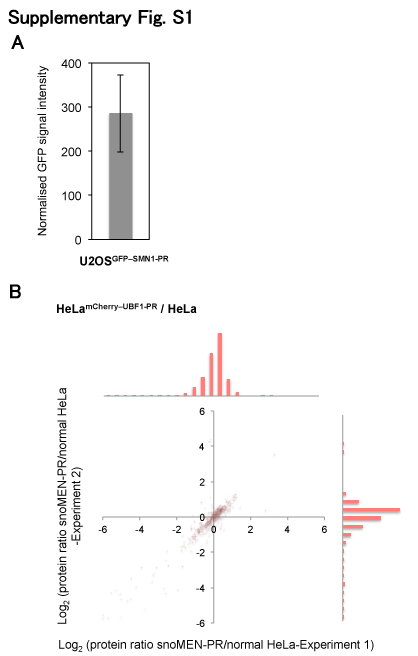

Supplement: Figure S1 — Gene-expression profile of snoMEN replacement stable cell lines. (A) Distribution pattern of GFP-SMN1 signal intensity of U2OSGFP–SMN1-PR stable cell line. Cytoplasmic GFP signals were calculated from randomly selected cells (n = 42). Each signal was normalised by DAPI signal. (B) Expression level comparison of proteins detected by Mass spectrometry for HeLamCherry–UBF1-PR versus HeLa cells. Each SILAC experiment was independently repeated at least three times. Correlation between protein ratios of SILAC experiments visualised on a 2D logarithmic graph for all proteins identified as previously demonstrated [48], [49]. On the x and y axis, log2 (H/L ratio) correlates with the enrichment in HeLamCherry–UBF1-PR versus HeLa cells for experiment 1 and experiment 2, respectively. Graph shows a distribution pattern of plot numbers. SILAC ratio values of labelled proteins are listed in Table S3. (TIF) [file pone.0062305.s001.tif]

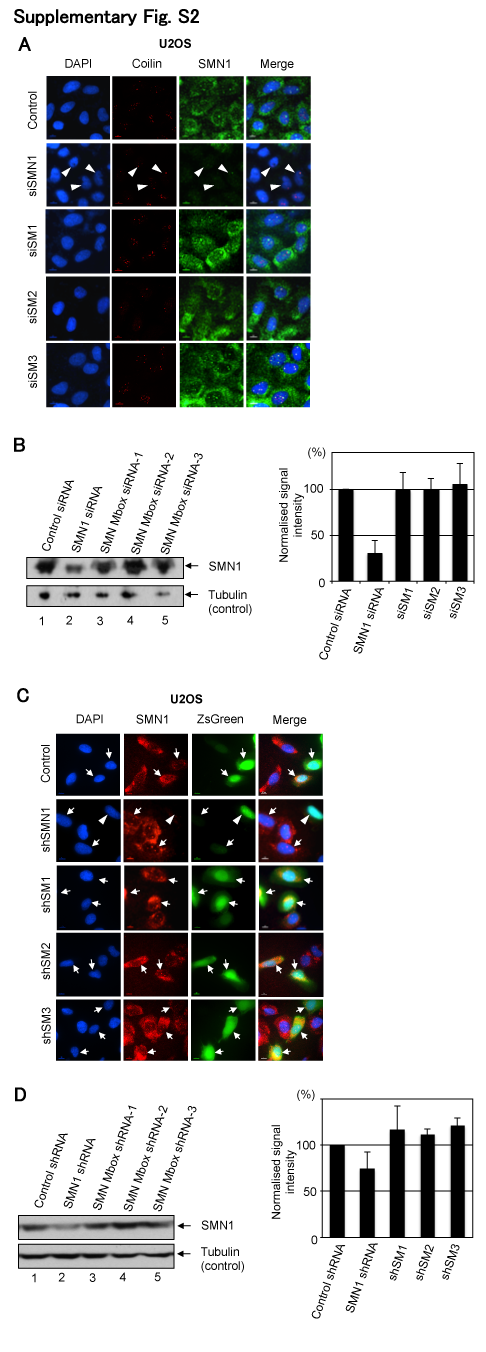

Supplement: Figure S2 — SiRNA and shRNA knock-down targeted to endogenous SMN1 pre-mRNAs. (A–D) These are the same experiment as in Figure 3C–F except the target gene is SMN1 in U2OS cells. (A) Scrambled siRNA (Negative control siRNA) and SMN1 siRNA (Dharmacon) were transfected as a negative and a positive control, respectively. SMN1 Mbox siRNA-1 to -3 (siSM1–3) have the same target sequence as SMN1 snoMEN from set1 to set3, respectively (Figure 3A). Scale bar, 10 µm. Arrowhead: cells showing knock-down. (B) Western blot analysis for siRNA experiments. Detection of protein levels for endogenous SMN1 following transfection of U2OS cells using either Scrambled siRNA (Control: lane1), SMN1 siRNA (siSMN1: lane2), SMN M box siRNA-1 (siSM1: lane3), SMN M box siRNA-2 (siSM2: lane4), and SMN M box siRNA-3 (siSM3: lane5). An equivalent amount of U2OS extract was loaded for each lane and the proteins separated by SDS PAGE, electroblotted onto membrane and probed both with a monoclonal anti-SMN1 antibody and with anti-tubulin as a loading control. Graph shows SMN1 signal intensity normalised to the tubulin signal measured from three independent experiments. (C) A shRNA plasmid targeted to SMN1 and no-endogenous target shRNA plasmid were transfected as a positive and negative control, respectively. SMN1 Mbox shRNA-1 to -3 (shSM1–3) have the same target sequence as SMN1 snoMEN from set1 to set3, respectively (Figure 3B). Scale bar, 10 µm. Arrow: cells not showing knock-down, Arrowhead: cells showing knock-down. (D) Western blot analysis for shRNA experiments. Detection of protein levels for endogenous SMN1 following transfection of U2OS cells using either Scrambled shRNA (Control: lane1), SMN1 shRNA (shSMN1: lane2), SMN M box shRNA-1 (shSM1: lane3), SMN M box shRNA-2 (shSM2: lane4), and SMN M box shRNA-3 (shSM3: lane5). An equivalent amount of U2OS extract was loaded for each lane and the proteins separated by SDS PAGE, electroblotted onto membrane and probed both with a monoclonal anti-SMN1 antibody a [file pone.0062305.s002.tif]

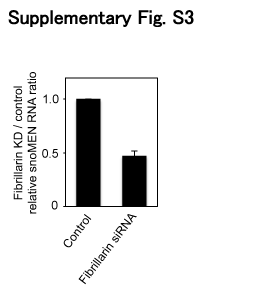

Supplement: Figure S3 — SnoRNA expression analysis after Fibrillarin knock-down treatment. qRT-PCR was performed to measure snoRNA expression level after treatment with scramble siRNA (Control) and Fibrillarin siRNA. Equal amounts of total RNA from U2OSGFP–SMN1-PR cells, extracted following siRNA treatment, was used for qRT-PCR reactions. Graph shows the snoRNA expression ratio between control and fibrillarin siRNA experiments measured from four independent experiments. SnoRNA HBII-180C (snoMEN backbone) specific primers and GAPDH mRNA specific primers, as a loading control, were used for amplification. (TIF) [file pone.0062305.s003.tif]

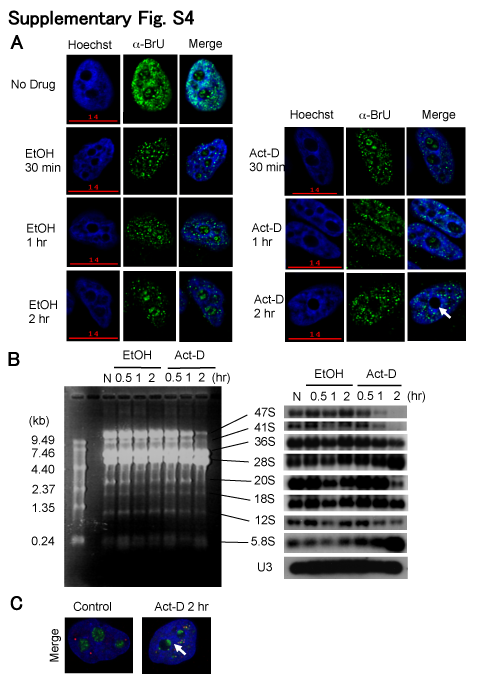

Supplement: Figure S4 — Optimisation of RNA polymerase I inhibition using low concentration Actinomycin-D treatment. (A) In vivo transcription assay with/without Actinomycin-D treatment in HeLa cells. HeLa cells were treated either with ethanol (EtOH) as a negative control, or with Actinomycin-D (0.01 µg/ml) for each time point: 30 min, 1 hr, and 2 hr. Transcription in the cells was detected via incorporation of 5-fluorouridine. Two hours following Actinomycin-D treatment, the nucleolar signal had disappeared (arrow). Scale bar indicates 14 µm. (B) Identification of pre-rRNA transcriptions. Northern blot analysis was performed to decide a time point of pre-rRNA inhibition by Actinomycin-D. Each pre-rRNA was detected by using probes specific to 5.8S, 18S, and 28S rRNAs. U3 snoRNA was also detected as a loading control. (C) Specific RNA polymerase I inhibition was confirmed by imaging fibrillarin and coilin localisation patterns. Fibrillarin accumulated only in nucleoli after low concentration Actinomycin-D treatment for 2 hr (arrow); however, the accumulation of coilin that should occur at the nucleolar cap on inhibition of RNA polymerases I, II and III with high concentration Actinomycin-D treatment (1 µg/ml) [28], was not seen. (TIF) [file pone.0062305.s004.tif]

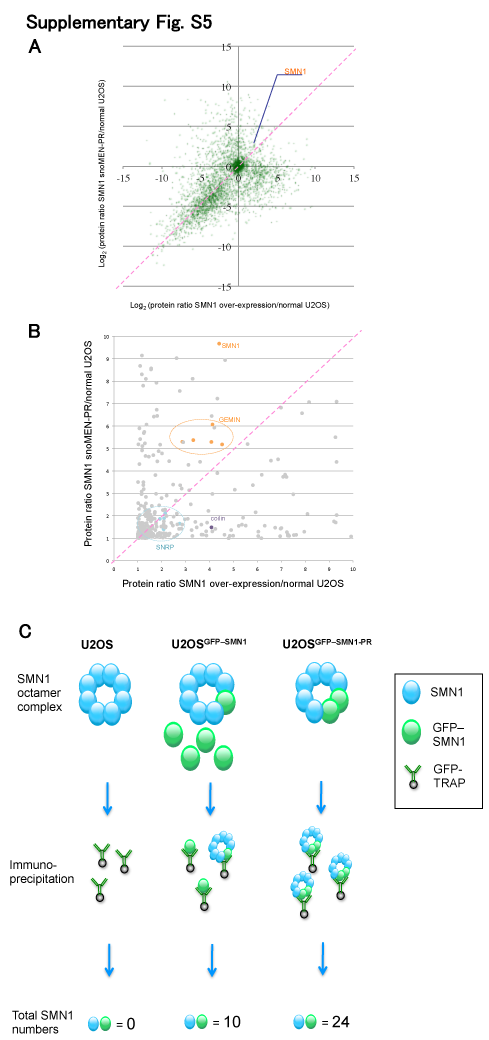

Supplement: Figure S5 — Characterisation of FP–protein complexes in replacement stable cell lines by Quantitative SILAC Proteomic analysis. (A) SILAC result of GFP–SMN1 complex pull-down assay visualised on a 2D logarithmic graph. On the x axis, log2 (H/L ratio) correlates with the enrichment in GFP–SMN1 IP with protein replacement versus control IP. On the y axis, log2 (M/L ratio) correlates with the enrichment in GFP–SMN1 IP versus control IP without replacement. The bait, SMN1, is highlighted in green, and a red line separates the proteins whose interaction with SMN1 is increased (above the line), or decreased (below) by protein replacement. SMN1 binding proteins which were identified and quantified are highlighted in B. SILAC ratio values of labelled proteins are listed in Table S4. (B) The graph [expanded top right segment of (a)] shows SILAC fold change ratio for known SMN binding proteins in the GFP–SMN1 IP. (C) The model of immuno–precipitation of endogenous SMN1 complex. (TIF) [file pone.0062305.s005.tif]
